# Triage and Allocation of Neurocritical Care Resources During the COVID 19 Pandemic - A National Survey

**DOI:** 10.3389/fneur.2020.609227

**Published:** 2021-01-06

**Authors:** Florian Gessler, Felix Lehmann, Julian Bösel, Hannah Fuhrer, Hermann Neugebauer, Katja E. Wartenberg, Stefan Wolf, Joshua D. Bernstock, Wolf-Dirk Niesen, Patrick Schuss

**Affiliations:** ^1^Department of Neurosurgery, University Hospital Frankfurt, Frankfurt, Germany; ^2^Department of Anesthesiology and Intensive Care Medicine, University Hospital Bonn, Bonn, Germany; ^3^Department of Neurology, Kassel General Hospital, Kassel, Germany; ^4^Department of Neurology, University Hospital Freiburg, Freiburg, Germany; ^5^Department of Neurology, University Hospital Würzburg, Würzburg, Germany; ^6^Department of Neurology, University Hospital Leipzig, Leipzig, Germany; ^7^Department of Neurosurgery, Charité University Hospital Berlin, Berlin, Germany; ^8^Department of Neurosurgery, Brigham and Women's Hospital, Boston, MA, United States; ^9^Harvard Medical School, Harvard University, Boston, MA, United States; ^10^Department of Neurosurgery, University Hospital Bonn, Bonn, Germany

**Keywords:** COVID-19, SARS-CoV, pandemic, patient triage, neurocritical care

## Abstract

**Objective:** In light of the ongoing COVID-19 pandemic and the associated hospitalization of an overwhelming number of ventilator-dependent patients, medical and/or ethical patient triage paradigms have become essential. While guidelines on the allocation of scarce resources do exist, such work within the subdisciplines of intensive care (e.g., neurocritical care) remains limited.

**Methods:** A 16-item questionnaire was developed that sought to explore/quantify the expert opinions of German neurointensivists with regard to triage decisions. The anonymous survey was conducted via a web-based platform and in total, 96 members of the Initiative of German Neurointensive Trial Engagement (IGNITE)-study group were contacted via e-mail. The IGNITE consortium consists of an interdisciplinary panel of specialists with expertise in neuro-critical care (i.e., anesthetists, neurologists and neurosurgeons).

**Results:** Fifty members of the IGNITE consortium responded to the questionnaire; in total the respondents were in charge of more than 500 Neuro ICU beds throughout Germany. Common determinants reported which affected triage decisions included known patient wishes (98%), the state of health before admission (96%), SOFA-score (85%) and patient age (69%). Interestingly, other principles of allocation, such as a treatment of “youngest first” (61%) and members of the healthcare sector (50%) were also noted. While these were the most accepted parameters affecting the triage of patients, a “first-come, first-served” principle appeared to be more accepted than a lottery for the allocation of ICU beds which contradicts much of what has been reported within the literature. The respondents also felt that at least one neurointensivist should serve on any interdisciplinary triage team.

**Conclusions:** The data gathered in the context of this survey reveal the estimation/perception of triage algorithms among neurointensive care specialists facing COVID-19. Further, it is apparent that German neurointensivists strongly feel that they should be involved in any triage decisions at an institutional level given the unique resources needed to treat patients within the Neuro ICU.

## Introduction

Faced with a potential second wave of the coronavirus disease-19 (COVID-19) Europe is again bracing for a potential resurgence of the virus driven in part by the liberalization of social distancing regulations (1). Unfortunately, intensive care unit (ICU) beds, ventilators, dialysis machines and personal protective equipment (PPE) have been and may continue to be scarce resources in regions with a high incidence of COVID-19.

As such, neurointensivists and their patients may face the prospect of rationing allocating valuable resources and in so doing be forced to triage patients when faced with overwhelming numbers COVID-19 patients in need of critical care (2). It is prudent to note that managing scarce medical resources/medically triaging patients is a foreign concept for most physicians throughout the Western world.

In order to determine triage criteria and possible algorithms for the allocation of limited ICU resources among neurointensivists, a survey was sent to members of the Initiative of German Neurointensive Trial Engagement (IGNITE)-study group in the early stages of the COVID-19 pandemic.

## Materials and Methods

Our COVID-19 triage survey was created in accordance with the Checklist for Reporting Results of Internet E-Surveys (CHERRIES) guidelines. The survey was sent to members of the Initiative of German Neurointensive Trial Engagement (IGNITE) consortium which consists of an interdisciplinary panel of specialists with expertise in neuro-critical care (i.e., anesthetists, neurologists and neurosurgeons) in March of 2020 prior to the initial wave of infections in Germany. Ninety-six neurointensivists were contacted via email. No incentives for participation in the survey were offered and those who refused to participate and/or did not complete the survey (i.e., more than three questions missing) were considered non-responders. The survey was available for a total duration of 3 weeks.

Briefly, the survey was developed by a multidisciplinary team whose members were from the Departments of Neurology, Neurosurgery and Anesthesiology & Intensive Care Medicine at the University Hospitals of Bonn, Frankfurt am Main and Freiburg. It was subsequently reviewed and revised based on feedback from other clinicians in terms of clarity, readability and content. Adaptive questioning was employed to reduce the complexity of the questions posed. In its final form, there were 1 to 2 questions per page and 12 pages in total. The anonymous survey was conducted via a web-based platform (SurveyMonkey Inc.; San Mateo, California, USA; www.surveymonkey.com). Unique visitors were identified based on IP addresses and were used to prevent multiple entries from the same individual. In cases of duplicate entries, the first entry was kept for analyses.

## Results

### Respondent Demographics

**Figure 1:**
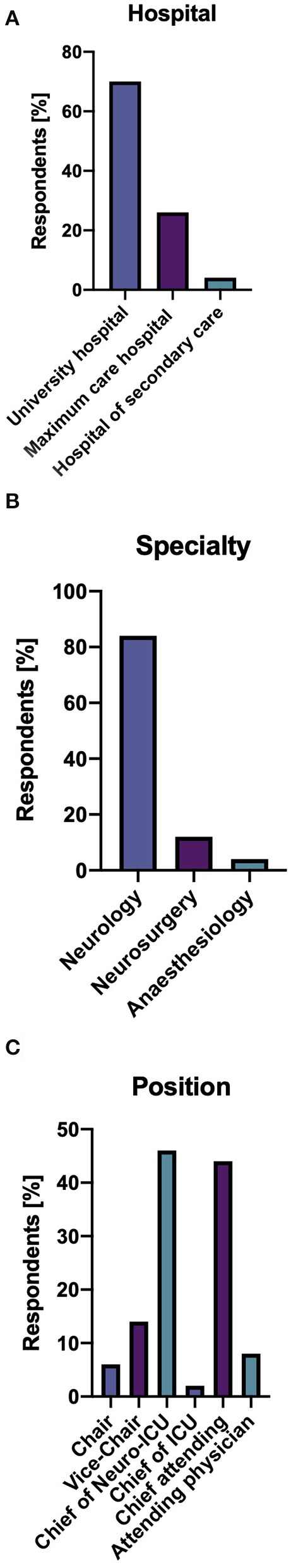
Characteristics of respondents. Bar charts of **(A)** type of institution, **(B)** respondent specialty, and **(C)** respondent position.

A total of 50 neurocritical care experts throughout Germany took part in the survey; yielding a response rate of ~ 55%. Seventy percentage of the respondents were employed at a University hospital, 26% at a hospital providing maximum academic care (>700 hospital beds), whereas 4% of respondents were employed at a hospital providing secondary care (500–700 hospital beds). Of the 50 neurointensivists who participated in the survey 84% specialized in neurology, 12% in neurosurgery and 4% in anesthesiology/critical care. Together, the respondents were responsible for a total of 519 Neuro ICU beds throughout Germany (Figure 1).

### Triage of Critical Care Resources

In the event that ICU beds were exhausted only 46% of the respondents recommended that decisions regarding the triage of resources for neuro-critical care patients be made by neurointensivists alone. However, the survey respondents also felt that at least one specialist in neuro-critical care should be an integral part of any central interdisciplinary triage team. Furthermore, the neurointensivists surveyed favored the involvement of local ethics committees should patient triage/diversion of resources be necessary.

Sixty-nine percentage of respondents felt that neurocritical resources should be made available to other critically ill patients thereby allowing for the admission and/or treatment of COVID-19 patients should the need arise. While the vast majority of polled neurointensivists appeared willing to share resources to help impacted areas (71%), many also felt that the needs of the local community the hospital was intended to serve should not be ignored (27%). In the case of limited but available ICU resources, 54% of respondents stated they would support the transfer of interstate and/or international patients for treatment, while 27% would recommend transferring critical patients from the vicinity of the hospital with higher priority.

When faced with hypothetical occupancy rate of 80% for ICU beds, the vast majority of respondents (78%) felt that the responsibility for the triage of patients should fall to an interdisciplinary team within the Emergency Department. With regard to patients already admitted to a Neuro ICU, 59% of the respondents felt that the triage of those patients should be managed a triage team guided by neurointensivists.

### Evaluation of Prioritization Principles

**Figure 2:**
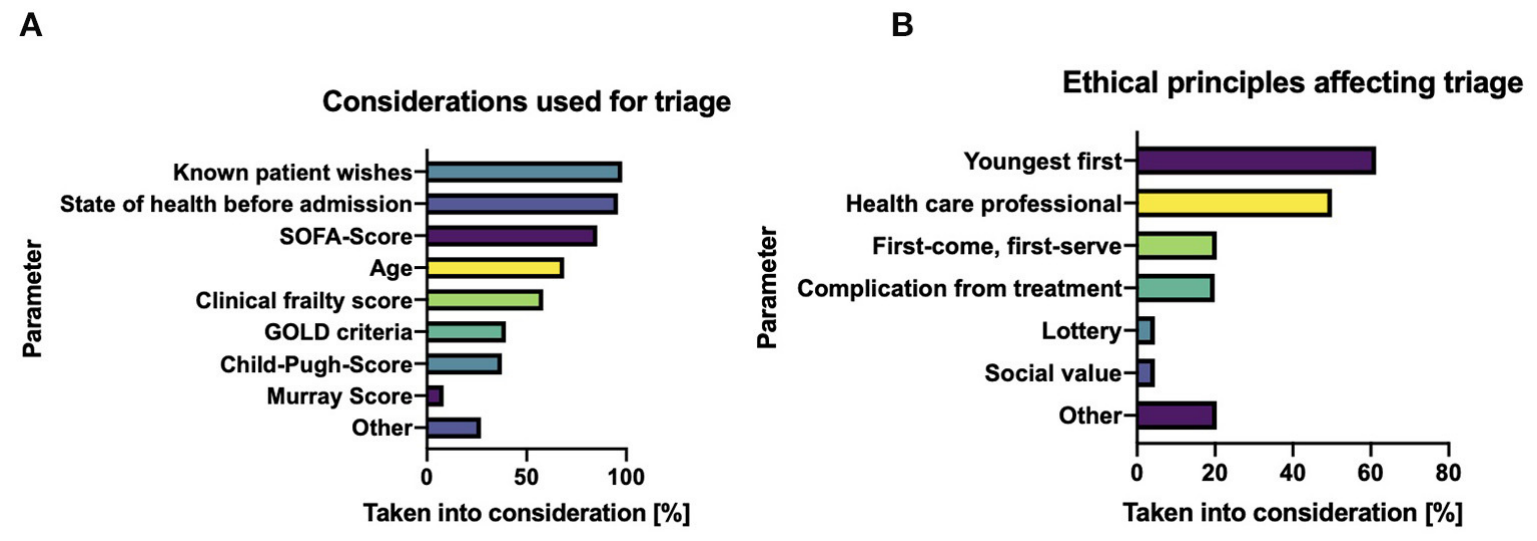
Tools for guiding triage decisions. **(A)** Determinants influencing triage at a stage of absolute intensive care scarcity. **(B)** Ethical principles of allocation during patient triage.

In the event that all ICU resources have been exhausted, patients within the ICU and/or scheduled for admission to the ICU may have to be triaged. The most important determinants affecting triage in this situation as per our survey were as follows: “known patient wishes” (98%), followed by “known state of health before acute deterioration” (96%), “SOFA score” (85%), and “patient age” (69%) (Figure 2A).

In addition, the majority of respondents also felt the following principles were important with regard to the triaging of ICU resources and should be weighed heavily: “youngest first” (61%), “patient is a health care worker” (50%), “first-come, first-serve” (20%), and “iatrogenic complications leading to ICU necessity” (16%). Unsurprisingly, respondents also felt that “worst short-time prognosis” and “results of daily visits by the ethics committee” were important factors to consider when making individual triage decisions (Figure 2B).

## Discussion

The work presented herein offers a valuable overview of patient triage from experts in a highly subspecialized field of intensive care medicine (3). Such work may have an immediate impact as Europe braces for a second wave of COVID-19 (1).

With a surge in infected patients suffering from COVID-19, patient triage with regard to ICU resources became necessary in various countries throughout Europe. Accordingly, a high degree of transparency is needed with regard to the principles, values, and criteria employed to facilitate such triage decisions is needed. While algorithms/tools have been proposed to facilitate the triage of critical ill patients, such an approaches have a myriad of shortcomings (4, 5). It is also prudent to note that while the information/values by which triage teams base their decisions are often abstract/imprecise they may have practical utility in that they serve to alleviate the moral distress/legal questions individual clinicians may face when triaging limited resources (6).

Critically the neurointensivists queried did not feel comfortable handing over responsibility for the patients they were taking care of, with the vast majority (78%) feeling that patient triage should be performed immediately upon presentation by an interdisciplinary team. Further when triaging for special-care ICU admission (i.e., neuro/neonatal), they felt that subspecialists should be an integral part of any interdisciplinary triage team. Such thoughts do not necessarily align with the work of Kirkpatrick et al. who have noted that to ensure an equitable distribution of resources, triage teams must be sheltered from the influence of factors that appear to be relevant only to the care of individuals (6). In line with such thinking the concept of allocation mandates that when medical resources are scarce, they must be used as efficiently as possible to achieve the greatest possible overall benefit (7). While recommendations on triage for viral pandemics and/or cases of (bio)terrorist attacks have been published, scarce data is available regarding the acceptance of such principles (8). Amongst such previously published principles of allocation, decisions took into account age (i.e., the “youngest first”) and exposure (i.e., “members of the health care sector”) seemed to be the most accepted parameters amongst the neurointensivists that were surveyed (6, 7).

It goes without saying that withdrawing healthcare resources (e.g., mechanical ventilation) from one party to give to another poses unparalleled stress on everyone that is involved in the process (i.e., triage teams, patients, physicians, relatives etc.) (7, 9). Of note, published guidelines do support the withdrawal of mechanical ventilation during certain public health crises, yet little work has described how such decisions would be practically implemented (9, 10). The results of our survey clearly illustrate that a variety of different tools, scores and/or metrics should be used to ensure that any resultant assessment is valid. Interestingly, a “first-come, first-serve” principle was accepted considerably more often (20%) than a “lottery system” (5%) with regard to patient triage. This opinion is notably discordant with the recent recommendations, which argue that the principle of “first-come, first-served” should not be employed (11). The thoughts of the neurointensivists surveyed appeared more aligned with the rule of absolute equality, as lottery system might lead to the discontinuation of treatment for a patient who may have a better chance of a meaningful outcome, even in the context of a “prognosis-matched lottery” (11). Further, the loss of physician input and decisional autonomy that would result from a lottery system may be untenable for many, including those within health care and society at large.

The COVID-19 pandemic has stretched many health systems to their very limits and beyond, and ultimately threatens to do so again if the pandemic reignites (1, 3). While decisions regarding the allocation of ICU resources will remain challenging it is the authors' hope/contention that our survey which quired individuals with regard to their thoughts/practice may ultimately relieve some of the individual burden shouldered by individual physicians throughout the world during such unprecedented times.

### Limitations

It is prudent to note that our study has several limitations. First and foremost, the present study was conducted via an online survey and was limited to members of the IGNITE consortium whose views may not necessarily be consistent with neurointensivists outside of Germany. Further, the survey sought only the neurointensivists which may differ dramatically from other critical care providers. In addition, this survey was based on – at that time - hypothetical circumstances in intensive care units. This might cause a different interpretation of the question resulting in divergent answers and thus, under certain circumstances, an erroneous interpretation. Accordingly, future studies that sample broader populations/subdisciplines of physicians/surgeons may ultimately be warranted. Despite such shortcomings, this survey does in fact represent the first assessment of triage behavior by neurointensivists in the face of the COVID-19 pandemic.

## Conclusions

The data presented within this survey highlight core tenants of patient triage paradigm as viewed by neurointensivists. Such work has made clear that neurointensivists feel that they should be involved in multidisciplinary triage team should ICU resources be exhausted in the face of a pandemic related patient surge.

## Data Availability Statement

The raw data supporting the conclusions of this article will be made available by the authors, without undue reservation.

## Author Contributions

FG, FL, W-DN, and PS: conception of the work/study, supervision, and drafting of the manuscript. JBö, HF, HN, KW, and SW: acquisition of data. FG, JBe, and PS: analysis/interpretation of the work and critical revision of the manuscript. All authors critically revised the work and gave final approval to the submitted version.

## Conflict of Interest

The authors declare that the research was conducted in the absence of any commercial or financial relationships that could be construed as a potential conflict of interest.

## References

[1] Kissler SM Tedijanto C Goldstein E Grad YH Lipsitch M. Projecting the transmission dynamics of SARS-CoV-2 through the postpandemic period. Science. (2020) 368:860–8. 10.1126/science.abb5793 32291278 PMC7164482

[2] Hulsbergen AFC Eijkholt MM Balak N Brennum J Bolger C Bohrer AM. Ethical triage during the COVID-19 pandemic: a toolkit for neurosurgical resource allocation. Acta Neurochir. (2020) 162:1485–90. 10.1007/s00701-020-04375-w 32405671 PMC7220806

[3] Truog RD Mitchell C Daley GQ. The toughest triage - allocating ventilators in a pandemic. N Engl J Med. (2020) 382:1973–5. 10.1056/NEJMp2005689 32202721

[4] Zivot J. Coronavirus disease 2019 triage teams: death by numbers. Crit Care Med. (2020) 48:1241–2. 10.1097/CCM.0000000000004435 32697500 PMC7365581

[5] Sprung CL Joynt GM Christian MD Truog RD Rello J Nates JL. Adult ICU triage during the coronavirus disease 2019 pandemic: who will live and who will die? recommendations to improve survival. Crit Care Med. (2020) 48:1196–202. 10.1097/CCM.0000000000004410 32697491 PMC7217126

[6] Kirkpatrick JN Hull SC Fedson S Mullen B Goodlin SJ. Scarce-resource allocation and patient triage during the COVID-19 Pandemic: JACC review topic of the week. J Am Coll Cardiol. (2020) 76:85–92. 10.1016/j.jacc.2020.05.006 32407772 PMC7213960

[7] White DB Lo B. A framework for rationing ventilators and critical care beds during the COVID-19 pandemic. JAMA. (2020) 323:1773–4. 10.1001/jama.2020.5046 32219367

[8] Rubinson L Nuzzo JB Talmor DS O'Toole T Kramer BR Inglesby TV. Augmentation of hospital critical care capacity after bioterrorist attacks or epidemics: recommendations of the working group on emergency mass critical care. Crit Care Med. (2005) 33:2393–403. 10.1097/01.CCM.0000173411.06574.D5 16215397

[9] Cohen IG Crespo AM White DB. Potential legal liability for withdrawing or withholding ventilators during COVID-19: assessing the risks and identifying needed reforms. JAMA. (2020) 323:1901–2. 10.1001/jama.2020.5442 32236491

[10] Piscitello GM Kapania EM Miller WD Rojas JC Siegler M Parker WF. Variation in ventilator allocation guidelines by US state during the coronavirus disease 2019 pandemic: a systematic review. JAMA Netw Open. (2020) 3:e2012606. 10.1001/jamanetworkopen.2020.12606 32558916 PMC7305526

[11] Emanuel EJ Persad G Upshur R Thome B Parker M Glickman A. Fair allocation of scarce medical resources in the time of covid-19. N Engl J Med. (2020) 382:2049–55. 10.1056/NEJMsb2005114 32202722

