# Supplementary material for: Triage and Allocation of Neurocritical Care Resources During the COVID 19 Pandemic - A National Survey
Source: Front Neurol. 2021 Jan 6;11:609227. doi: 10.3389/fneur.2020.609227 (PMC7874200; doi:10.3389/fneur.2020.609227)
Supplement: Supplementary file 1 [file Data_Sheet_1.docx]

**SUPPLEMENTARY APPENDIX**

**1. COLLABORATORS**

RWTH University Hospital Aachen, Germany

Anke Alexandra Alberty, MD

University Hospital Augsburg, Germany

Hauke Schneider, MD

Charité University Hospital Berlin, Germany

Julia Nichtweiss, MD

Farid Salih, MD

Bernd Vorderwülbecke, MD

LVR-Hospital Bonn, Germany

Christian Dohmen, MD

Patrick Merl, MD

University Hospital Bonn, Germany

Christian Bode, MD

Erdem Güresir, MD

Christian Putensen, MD

Matthias Schneider, MD

Hartmut Vatter, MD

Brigham and Women’s Hospital, Boston, USA

Omar Arnaout, MD

Ari D. Kappel, MD

Benjamin Johnston, MD, PhD

Saksham Gupta, MD

Jason A. Chen MD, PhD

Ian Tafel, MD

Henrikas Vaitkevicius, MD

University Hospital Cologne, Germany

Michael Malter, MD

Onur Özgür, MD

Henning Stetefeld, MD

Klinikum Darmstadt, Germany

Rainer Kollmar, MD

Ingo Schirotzek, MD

Klinikum Dortmund, Germany

Gernot Reimann, MD

University Hospital Erlangen, Germany

Joji Kuramatsu, MD

Hagen Huttner, MD

University Hospital Essen, Germany

Andreas Totzeck, MD

University Hospital Frankfurt, Germany

Waltraud Pfeilschifter, MD

Volker Seifert, MD

University Hospital Freiburg, Germany

Jürgen Bardutzky, MD

Jochen Brich, MD

University Hospital Göttingen, Germany

Ilko Maier, MD

University Hospital Hamburg-Eppendorf, Germany

Iris Lettow, MD

University Hospital Heidelberg, Germany

Silvia Schönenberger, MD

General Hospital Ingolstadt, Germany

Thomas Pfefferkorn, MD

University Hospital Jena, Germany

Albrecht Günther, MD

General Hospital Kassel, Germany

Jana Godau, MD

Gabor Nagy, MD

Christian Schmidt, MD

University Hospital Kiel, Germany

Johannes Meyne, MD

University Hospital Leipzig, Germany

Dominik Michalski, MD

Johann Pelz, MD

University Hospital Mainz, Germany

Thomas Kerz, MD

Peter Schramm, MD

University Hospital Mannheim, Germany

Angelika Alonso, MD

University Hospital Großhadern, LMU München, Germany

Andreas Bender, MD

Konstantinos Dimitriadis, MD

Matthias Klein, MD

University Hospital Münster, Germany

Rainer Dziewas, MD

Jens Minnerup, MD

Yale-New Haven Hospital, New Haven, USA

Jens Witsch, MD

University Medical Center Regensburg, Germany

Sylvia Bele, MD

Bernhard Neumann, MD

University Hospital Rostock, Germany

Matthias Wittstock, MD

University Hospital Saarland, Germany

Piergiorgio Lochner, MD

Niklas Kämpfer, MD

Wetzlar Clinic, Germany

Thorsten Schmelzer, MD

_______

**2. QUESTIONNAIRE (translated from German)**

1. Which state are you practicing in?

2. Which of the following best describes your hospital?

a. University hospital

b. Non-academic hospital of maximum care (>700 hospital beds)

c. Hospital of secondary-care (500-700 hospital beds)

d. General Hospital (300-500 hospital beds)

3. Which is your medical specialty?

a. Neurology

b. Neurosurgery

c. Anaesthesiology

d. Internal Medicine

4. What is your current position in your hospital?

a. Chair / Head of department

b. Vice chair

c. Chief of the neuro-intensive care unit

d. Chief of the intensive care unit

e. Senior physician

f. Attending physician

5. How many beds in neuro-intensive care are you responsible for?

6. What would you recommend doing with excess intensive care capacity?

a. Reserve capacity for potentially admitted critical patients of the local population

b. Provision of capacity for other/international patients after decision-making in each individual case

c. Declare capacities available to other/international patients in principle

d. No comment

7. What would you recommend doing with the available capacities in a specialized (e.g. neuro-) intensive care unit?

a. Reserve for critical patients with neurological disorders

b. Reserve for patients with Covid-19

c. Reserve for general intensive care patients to provide capacity for Covid-19 patients in other facilities

d. No comment

8. If 80% of your Neuro-ICU capacity is exhausted, who should triage patients in the emergency room?

a. Emergency department with interdisciplinary triage team

b. Neuro-ICU with specialists

9. If 80% of your Neuro-ICU capacity is exhausted, who should triage the patients on the Neuro-ICU?

a. Emergency department with interdisciplinary triage team

b. Neuro-ICU with specialists

10. If 100% of your Neuro-ICU capacity is exhausted, who should triage patients in the emergency room?

a. Emergency department with interdisciplinary triage team

b. Neuro-ICU with specialists

11. If 100% of your Neuro-ICU capacity is exhausted, who should triage the patients on the Neuro-ICU?

a. Emergency department with interdisciplinary triage team

b. Neuro-ICU with specialists

12. Given the limited capacity of the ICU, what criteria would you take into account when allocating patients to the ICU?

a. Patient age

b. SOFA-Score

c. Known patient wish

d. Known state of health before acute deterioration

e. Child-Pugh-Score

f. GOLD criteria

g. Clinical frailty scale

h. Murray score

i. Others - please comment

13. Given the scarce capacity of the ICU, what other criteria would you consider including in your patient triage?

a. Lottery

b. Iatrogenic complications leading to ICU necessity

c. Youngest first

d. Patient is a health care worker

e. Prioritization of patients with higher social value

f. First-come, first-serve

g. Others - please comment

14. What criteria would you include in your patient triage for a patient suffering from malignant middle cerebral artery infarction?

a. Left-sided localization of infarction

b. Age > 60 years

c. Known colonization with multi-resistant bacteria

d. Known dementia

e. Ischemic cardiomyopathy

15. What criteria would you include in your patient triage for a patient suffering from poor-grade subarachnoid hemorrhage (WFNS V)?

a. Ruptured aneurysm in the posterior circulation

b. Unilateral mydriasis

c. aortic valve disease with predominant aortic stenosis

d. Early signs of vasospasm-related infarction

e. Known malignant primary disease

16. What criteria would you include in your patient triage for a patient suffering from intracerebral hemorrhage?

a. Left-sided localization of hemorrhage

b. Age > 60 years

c. Presence of intraventricular hemorrhage

d. Liver cirrhosis Child-Pugh grade C

e. Patient under legal supervision with regard to health issues
